# Nodular Histologic Subtype and Ulceration are Tumor Factors Associated with High Risk of Recurrence in Sentinel Node-Negative Melanoma Patients

**DOI:** 10.1245/s10434-016-5566-8

**Published:** 2016-09-19

**Authors:** Marloes Faut, Kevin P. Wevers, Robert J. van Ginkel, Gilles F. H. Diercks, Harald J. Hoekstra, Schelto Kruijff, Lukas B. Been, Barbara L. van Leeuwen

**Affiliations:** 1 Departments of Surgical Oncology and Surgery, University Medical Center Groningen, University of Groningen, Groningen, The Netherlands; 2 Department of Pathology, University Medical Center Groningen, University of Groningen, Groningen, The Netherlands

## Abstract

**Background:**

Since its introduction, the sentinel lymph node biopsy (SLNB) has become the standard staging procedure in clinical node-negative melanoma patients. A negative SLNB, however, does not guarantee a recurrence-free survival. Insight into metastatic patterns and risk factors for recurrence in SLNB negative melanoma patients can provide patient tailored guidelines.

**Methods:**

Data concerning melanoma patients who underwent SLNB between 1996 and 2015 in a single center were prospectively collected. Cox regression analyses were used to determine variables associated with overall recurrence and distant first site of recurrence in SLNB-negative patients.

**Results:**

In 668 patients, SLNBs were performed between 1996 and 2015. Of these patients, 50.4 % were male and 49.6 % female with a median age of 55.2 (range 5.7–88.8) years. Median Breslow thickness was 2.2 (range 0.3–20) mm. The SLNB was positive in 27.8 % of patients. Recurrence rates were 53.2 % in SLNB-positive and 17.9 % in SLNB-negative patients (*p* < 0.001). For SLNB-negative patients, the site of first recurrence was distant in 58.5 %. Melanoma located in the head and neck region (hazard ratio 4.88, *p* = 0.003) and increasing Breslow thickness (hazard ratio 1.15, *p* = 0.013) were predictive for distant first site of recurrence in SLNB-negative patients. SLNB-negative patients with a nodular melanoma and ulceration had a recurrence rate of 43.1 %; the site of recurrence was distant in 64 % of these patients.

**Conclusions:**

The recurrence rates of SLNB-negative nodular ulcerative melanoma patients approach those of SLNB-positive patients. Stringent follow-up is recommended in this subset of patients.

Cutaneous melanoma mainly spreads via the lymphogenic route from the sentinel lymph nodes to the adherent lymph node basin. After decades of experience, we now know that in negative sentinel lymph node biopsy (SLNB), skip metastases are rare.^1^,^2^ Since its introduction in the early 1990s by Donald Morton, SLNB has become a widely accepted staging procedure and has become one of the most important prognostic tools in predicting outcome in cutaneous melanoma.^3^–^7^ At time of primary staging, approximately 20 % of melanoma patients are SLNB positive, with a false-negative rate less than 5 %.^1^,^8^–^10^

SLNB positivity is associated with several clinicopathologic characteristics, such as Breslow thickness, mitosis, and the presence of ulceration.^11^–^13^ The risk for recurrent disease is associated with this SLNB status, resulting in higher recurrence rates of up to 47 % in SLNB-positive patients and lower recurrence rates of 24 % in SLNB-negative patients.^14^–^17^ Some of these SLNB-negative patients have a distant first site of recurrence and even seem to skip the lymphogenic metastatic route.^15^ These patients with direct hematogenous recurrences may either have different clinicopathologic characteristics or more aggressive tumor biology.

The purpose of this study was to evaluate risk factors for recurrent disease and distant first site of recurrence in SLNB-negative melanoma patients.

## Methods

The study population consisted of all consecutive melanoma patients who underwent a wide excision with a 1–2 cm margin according to the international melanoma guidelines and a SLNB at the University Medical Center Groningen (UMCG) between 1996 and 2015. The SLNB technique used at the UMCG has been described elsewhere in detail.^18^ Patient- and tumor-related clinicopathologic characteristics were prospectively collected in a database. Data concerning patient and tumor clinicopathologic characteristics, follow-up, recurrence, and survival were retrieved from the database for analysis.

Statistics were performed by IBM SPSS 22.0 (IBM SPSS, Chicago, IL, USA). Differences between groups were analyzes by the Chi square test for nominal variables; for continuous variables, the one-way ANOVA or Kruskal–Wallis test was used. Cox regression analyses were used to determine variables associated with overall recurrence in all patients and distant first site of recurrence in SLNB-negative patients. Overall recurrence was defined as any recurrence besides recurrence in the same basin as the SLNB. On the basis of our data, the following were included in the analysis: patient demographics, histologic type, location of primary lesion, Breslow thickness, Clark level, ulceration, mitosis, regression, lymphangioinvasion, use of immunosuppressant medication, and whether the primary excision was radical. Variables were checked for correlation with Pearson’s or Spearman’s rho. Variables on a 20 % significance level in the univariate Cox regression were entered in the multivariate Cox regression analysis. In the multivariate analysis, variables were checked for multicollinearity and confounding. Confounding limit was set at 10 %. Confounders and variables with a multicollinear association were excluded from multivariate analysis. Variables with *p* < 0.05 in the multivariate analysis were identified as significant factors.

Melanoma-specific survival (MSS), disease-free survival (DFS), and time to death from moment of first recurrence were analyzed by the Kaplan–Meier test. SLNB was defined as falsely negative if the first site of recurrence was in the same basin as the SLNB, and also when combined with systemic recurrence. To determine whether a SLNB was falsely negative in case of systemic recurrence, all positron emission tomography/computed tomography scans performed at the moment of systemic recurrence were reviewed to check for nodal involvement in the same basin as the SLNB. Because of the 100 % recurrence rate in the same basin in falsely negative SLNB patients, they were not included in the Cox regression analysis.

In the case of multisite recurrence, the recurrence site with the worst prognosis was scored as the first site of recurrence. For example, in the case of recurrence in the retroperitoneal lymph nodes and brain metastases, brain metastases were scored as the first site of recurrence. Follow-up was conducted in the UMCG. We received institutional review board approval, and the study was conducted according to the declaration of Helsinki.

## Results

During the study period, a SLNB was performed in 668 patients. Baseline clinicopathologic characteristics of all patients are displayed in Table 1. Median (range) age at diagnosis of primary melanoma was 55.2 (5.7–88.8) years, and superficial spreading melanoma was the most common histologic subtype (62 %). Median overall Breslow thickness was 2.2 (0.30–20.0) mm. The median Breslow thickness in the different histologic subtypes was as follows: superficial spreading melanoma (*n* = 414), 1.8 (0.30–9.0) mm; nodular melanoma (*n* = 192), 3.4 (0.9–20) mm; acral lentiginous melanoma (*n* = 21), 3.6 (1.1–11) mm; other melanomas (*n* = 28), 3.3 (0.85–17.00) mm; and unknown histologic subtype (*n* = 13), 3.00 (1.0–7.0) mm. SLNB was positive in 27.8 % of patients. In SLNB-negative patients, 24 patients experienced a nodal recurrence in the same basin as the SLNB, resulting in a SLNB false-negative rate of 3.6 %. In Table 1, the differences between the baseline clinicopathologic characteristics are displayed by SLNB status. During the median follow-up of 58.8 (range 1.8–190) months, a recurrence was diagnosed in 82 of the truly SLNB-negative patients (17.9 %) and in 99 SLNB-positive patients (53.2 %).

**Table 1:** Clinicopathologic characteristics overall, in SLNB-negative patients, and in SLNB-positive patients (*n* = 668)

Characteristic	Overall (*n* = 668)	SLNB negative (*n* = 458)	SLNB positive (*n* = 186)	*p*
Sex				0.070
Male	337 (50.4)	220	104	
Female	331 (49.6)	238	82	
Age, years^a^	55.2 (5.7–88.8)	55.3 (11.5–88.8)	53.5 (5.7–88.8)	0.890
Site of primary lesion				0.003
Lower extremity	228 (34.1)	149	68	
Head and neck	95 (14.2)	75	16	
Trunk	256 (38.3)	166	86	
Upper extremity	89 (13.3)	68	16	
Histologic typing				0.705
Superficial spreading	414 (62)	291	110	
Nodular	192 (28.7)	128	56	
Acral lentiginous	21 (3.1)	12	7	
Other^b^	28 (4.2)	20	8	
Breslow thickness, mm	2.2 (0.30–20.0)	1.9 (0.3–20.0)	3.00 (0.8–13.0)	<0.001
T stage				<0.001
T1 (<1.00 mm)	38 (5.7)	7	0	
T2 (1.01–2.00 mm)	271 (40.6)	155	34	
T3 (2.01–4.00 mm)	244 (36.5)	114	83	
T4 (>4.00 mm)	114 (17.1)	52	37	
Clark level				0.035
II	7 (1.0)	5	2	
III	137 (20.5)	109	26	
IV	472 (70.7)	312	141	
V	40 (6.0)	23	14	
Ulceration				0.001
Yes	223 (33.4)	133	80	
No	435 (65.1)	319	103	
Mitosis				0.055
Yes	561 (84)	380	159	
No	45 (6.7)	37	7	

Data are presented as *n* (%) or median (range)

*SLNB* sentinel lymph node biopsy

^a^ Age at diagnosis of primary melanoma

^b^ Other histologic types are verrucous, spitzoid, epithelioid, desmoplastic melanoma, and lentigo maligna melanoma

Multivariate Cox regression analysis revealed the following variables to be associated with overall recurrence in SLNB-negative patients: male sex (hazard ratio [HR] 1.78, *p* = 0.025), increasing age (HR 1.02, *p* = 0.0085) per year, melanoma located in the head and neck region (HR 2.16, *p* = 0.024), nodular melanoma (HR 1.82, *p* = 0.028), and presence of ulceration (HR 2.11, *p* = 0.002). In SLNB-positive patients, excisional biopsy decreased the risk for recurrence (HR 0.49, *p* = 0.005) as well as melanoma located on the upper extremity (HR 0.37, *p* = 0.045). Male sex (HR 1.10, *p* = 0.048), increasing Breslow thickness (HR 1.09, *p* = 0.048), and the presence of ulceration (HR 2.15, *p* < 0.001) were associated with recurrence in this group. Mitosis and Clark level were not included in both multivariate analyses because of multicollinearity with Breslow thickness (Table 2). In SLNB-negative patients with a nodular melanoma, the recurrence rate was 38 (29.7 %) of 128; if ulceration was also present in the primary melanoma, the recurrence rate was increased to 43.1 %. The site of recurrence was distant in 64 % of these patients. Of all SLNB-negative patients, 12.7 % had nodular ulcerated melanoma. In the entire group of SLNB-negative patients with nodular melanoma, 25 % eventually progressed to distant disease, 34.5 % if ulceration was also present.

**Table 2:** Univariate and multivariate Cox regression analysis of clinicopathologic characteristics associated with overall recurrence in all patients and by SLNB status (*n* = 668)

Characteristic	Recurrence overall (*n* = 205)	Univariate SLNB (*n* = 82)		Multivariate SLNB negative			Univariate SLNB positive (*n* = 99)		Multivariate SLNB positive		
		HR	*p*	HR	*p*	95 % CI	HR	*p*	HR	*p*	95 % CI
Sex											
Male	126 (61.5)	2.34	<0.001*	1.78	0.025	1.08–2.94	1.34	0.156*	1.10	0.048*	1.01–1.20
Female	79 (38.5)										
Age^a^	58.66 (19.2–81.4)	1.03	0.001*	1.02	0.008*	1.01–1.04	1.02	0.036*	1.01	0.127	0.99–1.03
Site of primary lesion											
Lower extremity	71 (34.6)	1.00	0.010*	1.00	0.006*		1.00	0.100	1.00	0.035*	
Head/neck	34 (16.6)	2.20	0.011*	2.16	0.024*	1.11–4.21	1.48	0.264	1.79	0.144	0.82–3.92
Trunk	84 (41)	1.47	0.156	1.34	0.346	0.73–2.64	0.93	0.600	0.89	0.687	0.53–1.52
Upper extremity	16 (7.8)	0.56	0.206	0.43	0.068	0.17–1.07	0.39	0.046*	0.37	0.045*	0.14–0.98
Histologic typing											
Superficial spreading	106 (51.7)	1.00	0.002*	1.00	0.164		1.00	0.840			
Nodular	74 (36.1)	2.50	<0.001*	1.82	0.028*	1.07–3.09	0.91	0.669			
Acral lentiginous	11 (5.4)	3.45	0.019	2.76	0.080	0.89–8.59	1.46	0.418			
Other^b^	10 (4.9)	1.82	0.257	1.02	0.978	0.30–3.50	1.31	0.534			
Excision radical											
Yes	153 (74.6)	0.93	0.786				0.49	0.001*	0.49	0.005*	0.30–0.81
No	52 (25.4)										
Breslow thickness, mm	3.00 (1.05–20.00)	1.16	<0.001*	1.06	0.151	0.98–1.16	1.11	0.007*	1.09	0.048*	1.00–1.20
Clark level											
II	1 (0.5)	1.91	0.524								
III	27 (13.2)	0.79	0.443				0.60	0.133			
IV	150 (73.2)	1.00	0.042*				1.00	0.311			
V	23 (11.2)	2.39	0.012*				1.39	0.333			
Ulceration											
Yes	110 (53.7)	3.02	<0.001*	2.11	0.002*	1.31–3.39	2.28	<0.001*	2.15	<0.001*	1.40–3.29
No	92 (44.9)										
Mitosis											
Yes	178 (86.8)	5.58	0.088				2.32	0.240			
No	4 (2.0)										
Regression											
Yes	22 (10.7)	0.99	0.840				0.97	0.206			
No	111 (54.1)										
Lymphangioinvasion											
Yes	18 (8.8)	1.14	0.081	1.16	0.172	0.94–1.43	0.92	0.641			
No	184 (89.8)										
Immunosuppressant medication											
Yes	7 (3.4)	2.37	0.143				1.12	0.819	1.97	0.193	0.71–5.43
No	198 (96.6)										

Data are presented as *n* (%) or median (range)

*SLNB* sentinel lymph node biopsy, *NA* not applicable

* *p* < 0.05. All variables with significance level of *p* < 0.2 in univariate Cox regression analysis were entered into multivariate Cox regression analysis

^a^ Age at diagnosis of primary melanoma

^b^ Other histologic types are verrucous, spitzoid, epithelioid, desmoplastic melanoma, and lentigo maligna melanoma

Table 3 shows the distribution of recurrence patterns for both SLNB-negative and -positive patients. The most common site of first recurrence was distant in all SLNB categories. In SLNB-negative patients, this was 58.5 % of all first recurrence sites. Of all 181 patients with recurrence, 77 % developed overall distant disease at some point in the course of their disease. If patients progressed to stage IV disease during the course of their disease, the largest portion of these distant recurrences was American Joint Committee on Cancer stage M1c (82.5 %).

**Table 3:** Recurrence rates and site of first recurrence in SLNB-negative and -positive patients

Characteristic	Overall, *n* (%)	SLNB negative, *n*	SLNB positive, *n*	*p*
Recurrence				<0.001
Yes	205 (30.7)	82	99	
No	463 (69.3)	376	87	
Type of first recurrence				0.053
Locoregional	30 (14.7)	14	16	
In transit	43 (21.1)	18	25	
Basin of SLNB/CLND	30 (14.7)	0	9	
Lymphatic	3 (1.5)	1	2	
Distant	98 (48)	49	46	
M-stage distant recurrence				0.278
M1a	4 (4.1)	3	1	
M1b	12 (12.4)	4	8	
M1c	80 (82.5)	42	37	
Unknown	1			

*SLNB* sentinel lymph node biopsy, *CLND* completion lymph node dissection

**Fig. 1:**
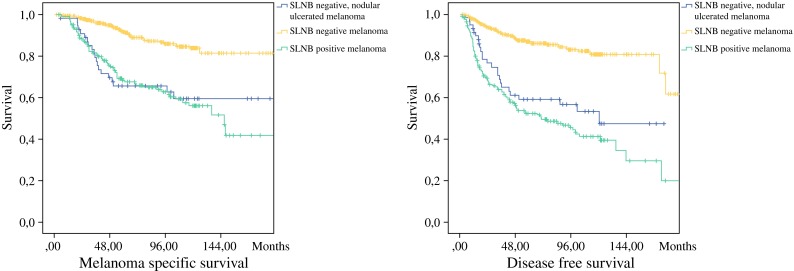
Survival split by sentinel lymph node biopsy negativity or positivity with nodular subtype and ulceration. **a** Melanoma-specific survival. **b** Disease-free survival

MSS and DFS was significantly worse for SLNB-positive patients compared to SLNB-negative patients (*p* < 0.001). If a recurrence had occurred, survival did not differ between SLNB-negative and SLNB-positive patients. There was a significant difference between MSS and DFS in SLNB-negative patients with ulcerated and nodular melanoma compared to the overall SLNB-negative group (*p* < 0.001; Fig. 1).

Multivariate Cox regression analysis revealed melanomas located on the head and neck (HR 4.88, *p* = 0.003), trunk (HR 3.33, *p* = 0.012), and upper extremity (HR 6.60, *p* = 0.008) to be associated with distant first site of recurrence in SLNB-negative melanoma patients as well as increasing Breslow thickness (HR 1.15, *p* = 0.013). The absence of mitosis (HR 0.06, *p* = 0.035) is protective for distant first site of recurrence in SLNB-negative patients (Table 4).

**Table 4:** Univariate and multivariate Cox regression analysis of clinicopathologic characteristics associated with distant first site of recurrence in SLNB-negative patients

Characteristic	Distant first recurrence (*n* = 48)	Univariate		Multivariate		
		HR	*p*	HR	*p*	95 % CI
Sex						
Male	32 (66.7)	1.43	0.243			
Female	16 (33.3)					
Age, y^a^	60.2 (19.4–79.6)	1.03	0.009*	1.02	0.160	0.99–1.04
Site of primary lesion						
Lower extremity	11 (22.9 %)	1.00	0.113	1.00	0.011*	
Head/neck	13 (27.1)	2.79	0.015*	4.88	0.003*	1.74–13.73
Trunk	20 (41.7)	1.68	0.169	3.33	0.012*	1.31–8.48
Upper extremity	4 (8.3)	1.68	0.374	6.60	0.008*	1.63–26.74
Histologic typing						
Superficial spreading	19 (39.6)	1.00	0.124	1.00	0.103	
Nodular	24 (50)	2.06	0.022*	1.91	0.079	0.93–3.93
Acral lentiginous	2 (4.2)	2.32	0.266	2.05	0.437	0.34–12.54
Other^b^	3 (6.3)	1.18	0.795	0.13	0.107	0.01–1.56
Breslow thickness, mm	3.00 (1.05–20.0)	1.12	0.008*	1.15	0.013*	1.03–1.29
Clark level						
II	0					
III	9 (18.8)	1.59	0.650			
IV	29 (60.4)	1.00	0.581			
V	7 (14.6)	0.77	0.548			
Missing	3 (6.2)					
Ulceration						
Yes	27 (41.7)	1.42	0.231			
No	20 (56.3)					
Missing	1 (2.1)					
Mitosis						
Yes	42 (87.5)					
No	1 (2.1)	0.03	0.004*	0.06	0.035*	0.01–0.82
Missing	5 (10.4)					
Regression						
Yes	21 (43.8)	0.99	0.777			
No	7 (14.6)					
Unknown	20 (41.7)					
Lymphangioinvasion						
Yes	4 (8.3)	1.03	0.766	1.04	0.880	0.63–1.71
No	43 (89.6)					
Immunosuppressant medication						
Yes	1 (2.1)					
No	47 (97.9)	0.82	0.843			

Data are presented as *n* (%) or median (range)

*SLNB* sentinel lymph node biopsy

* *p* < 0.05. All variables with significance level of *p* < 0.2 in univariate Cox regression analysis were entered into multivariate Cox regression analysis

^a^ Age at diagnosis of primary melanoma

^b^ Other histologic types are verrucous, spitzoid, epithelioid, desmoplastic melanoma, and lentigo maligna melanoma

## Discussion

The current study revealed that in SLNB-negative patients, recurrence rates approach the recurrence rates of SLNB-positive patients (43.1 vs. 53.2 %) if the unfavorable variables nodular histologic subtype and ulceration are accounted for. These pathologic characteristics are present in 12.7 % of SLNB-negative melanoma patients. As displayed by the Kaplan–Meier curves in Fig. 1, DFS and MSS is significantly worse for SLNB-negative nodular and ulcerated melanoma patients compared to the overall SLNB-negative group.

Risk factors for recurrent disease in SLNB-negative patients were increasing age, male sex, melanoma located on the head and neck region, nodular melanoma, and presence of ulceration (Table 2). Except for nodular melanoma, the significance of these variables in predicting recurrent disease in SLNB-negative melanoma patients was previously illustrated by several authors.^17^,^19^,^20^ O’Connell et al. recently stated that nodular histologic subtype approached significance in predicting recurrence in SLNB negative melanoma patients.^17^ Obviously a negative SLNB might create the impression of a less aggressive melanoma; however, one cannot be so sure when it concerns a nodular subtype. Because the recurrence percentage of SLNB-negative patients with ulcerated nodular melanoma approaches the recurrence percentage of SLNB-positive melanoma patients, primary tumor characteristics are apparently more relevant for recurrence in these patients than the status of the sentinel node. In absence of a lymphogenous recurrence and/or positive SLNB, risk factors for distant first site of recurrence were melanoma located on the head and neck, increasing Breslow thickness, and the presence of ulceration. In SLNB-negative patients, distant first site of recurrence occurred in 58.8 % of all recurrences. In this subset of patients, the melanoma seems to skip the lymphogenic metastatic route and metastasizes directly hematogenously. Overall, it is expected that unfavorable tumor clinicopathologic characteristics should be more frequent in the SLNB-positive group.^6^,^12^,^13^,^21^ Previous publications on SLNB-negative patients revealed increasing Breslow thickness, ulceration, head and neck melanoma, and unexpected lymph drainage patterns to be predictors for distant recurrence.^14^–^16^,^19^,^20^ Unexpected or aberrant lymph drainage patterns are expected in head and neck melanomas more than melanomas located on the upper and lower extremity. An affinity for hematogenous spread is suggested in this subset of melanomas.^22^,^23^ This might be an explanation for our findings.

The recurrence percentages were increased by more than twofold in the group of SLNB-negative patients with a nodular ulcerated melanoma compared to the whole group, suggesting a higher likeliness of hematogenous spread in these patients. Morton et al. posed two dissemination theories: the incubator hypothesis and the marker hypothesis. According to the first hypothesis, melanoma metastasizes mostly to the lymph nodes and in approximately 10 % directly via the hematogenous route. Tumor cells may grow in the SLNB but might incubate before spreading to distant sites. Removal of the SLNB and adherent lymph nodes can prevent further spread. The marker hypothesis, however, implicates a simultaneous spread. Tumor load in the SLNB is merely a marker for the ability of the tumor to spread. According to both hypotheses, the absence of melanoma cells in the SLNB indicates a primary melanoma unlikely to disseminate to distant sites.^24^ Perhaps the 10 % direct distant spread posed in the incubator hypothesis is caused by melanomas with unfavorable prognostics such as nodular subtype and ulceration. This hematogenous dissemination route was also described by Gershenwald et al., who suggested a subset of patients with a pure hematogenous dissemination route, without nodal involvement.^16^ Nodular melanoma is usually detected at a higher Breslow thickness than superficial spreading melanoma, even though the duration of change in a lesion before treatment is shorter in nodular melanoma than the superficial spreading type, which is suggestive for aggressive biologic behavior.^25^ Lin et al. published results where a significantly lower amount of tumor-infiltrating lymphocytes were found in nodular melanoma compared to superficial spreading melanoma, suggesting a different immunogenicity between the different histologic subtypes.^26^ Unfortunately, we do not routinely look for tumor-infiltrating lymphocytes in our institution, so we were not able to cross-reference this to our data.

The presented data on increased recurrence rates in patients with ulcerated nodular melanoma, increasing Breslow thickness melanoma, and head and neck localization are crucial for clinicians involved in melanoma care, as these findings can not only alter decisions on the duration and frequency of follow-up but also increase the awareness of the likelihood of distant recurrence in these patients. Therefore, we propose to distinguish a high risk for recurrence in the SLNB-negative subgroup.

SLNB-positive patients have a worse DFS compared to SLNB-negative patients. However, when a recurrence occurs, survival is similar. Survival was shorter in patients with a distant first site of recurrence compared to other recurrent sites, which has been extensively described in the literature.^15^,^27^

Patients with unfavorable primary clinicopathologic characteristics such as positive SLNB are already considered for adjuvant targeted therapy or immunotherapy trials. It might also be worth considering high-risk SLNB-negative patients for inclusion. First, however, adjuvant studies have yet to show a beneficial effect in SLNB-positive patients with respect to MSS. Before expanding inclusion criteria, the risk–benefit ratio should be properly assessed.

Although many researchers have focused on recurrence patterns of melanoma, we are still unable to accurately predict the course of the disease in many patients. We believe that most of the biological behavior in the end can be explained by possible unrevealed genetic mutations with distinctive biological behavior. Today, melanoma is characterized and staged by clinicopathologic features. There might be a role for genetic profiling aiming to identify melanoma patients with a misleadingly favorable SLNB pathologic prognosis whose disease is likely to recur in the future.
